# Atypical symptoms of testicular torsion in a 7-year-old child: A case report

**DOI:** 10.1097/MD.0000000000033709

**Published:** 2023-05-05

**Authors:** Bei-Cyuan Guo, Han-Ping Wu

**Affiliations:** a Department of Pediatrics, National Cheng Kung University Hospital, College of Medicine, National Cheng Kung University, Tainan, Taiwan; b College of Medicine, Chang Gung University, Taoyuan, Taiwan; c Department of Pediatrics, Chiayi Chang Gung Memorial Hospital, Chiayi, Taiwan.

**Keywords:** abdominal pain, atypical symptoms, testicular pain, testicular torsion

## Abstract

Testicular torsion accounting for 25% of acute scrotal disease, is an acute surgical condition. Atypical presentations of testicular torsion may lead delay diagnosis.

**Patient concerns:**

A 7-year-old boy was admitted to the pediatric emergency department with continuous and progressive left scrotal pain for 2 days, associated symptoms and signs included left scrotal swelling and erythema. The pain started 4 days ago as left lower abdominal pain which then migrated to the left scrotum.

**Diagnoses:**

Physical examination showed left scrotum skin redness, swelling, local heat, tenderness, high-riding testis, absence of the left side cremasteric reflex and a negative Prehn’s sign. Subsequent point of care ultrasound of scrotum revealed increased volume of the left testicle, inhomogeneous hypo-echoic left testis, and no detectable flow in the left testis. Left testicular torsion was diagnosed.

**Interventions:**

Surgical examination confirmed testicular torsion showing 720° counterclockwise rotation of the spermatic cord with ischemic changes in the left testis and epididymis.

**Outcomes:**

The patient was stabilized and discharged after left orchiectomy, right orchiopexy and antibiotic therapy.

**Lessons:**

Symptoms of testicular torsion may be atypical, especially in prepubertal age. Detailed history, physical examination, point of care ultrasound usage and timely urologist consultation and intervention are important for prompt rescue to prevent testicular loss, testicular atrophy, and eventual impairment of fertility.

## 1. Introduction

Acute pediatric scrotal pain often requires prompt surgical intervention.^[1]^ The most common causes of acute scrotal pain in children and adolescents include testicular torsion, torsion of the appendix testis, and epididymitis. In some studies, torsion of testis accounted for up to 25% of acute scrotal diseases in pediatrics and could be considered as a critical emergency condition.^[2]^ The annual incidence of testicular torsion is approximately 1 per 26,000 in the pediatric population.^[3]^ It presents with a bimodal age distribution, with 1 peak in the perinatal period, and the second peak around adolescence.^[3,4]^ Testicular torsion can be present in outside the common age of occurrence and an atypical pattern.^[5]^ Although rare, bilateral testicular torsion can happen in the pediatrics.^[6,7]^ Additionally, atypical presentations with abdominal pain, groin pain, nausea, and torsion in the abdomen or inguinal area may cause misdiagnosis or delay diagnosis.^[8,9]^ Testicular torsion is an urgent condition that can lead to testicular loss, and eventual impairment of fertility which makes prompt surgical exploration and treatment essential.^[10]^ This case reports an atypically presenting prepubertal male with testicular torsion.

## 2. Case presentation

**Figure 1.**
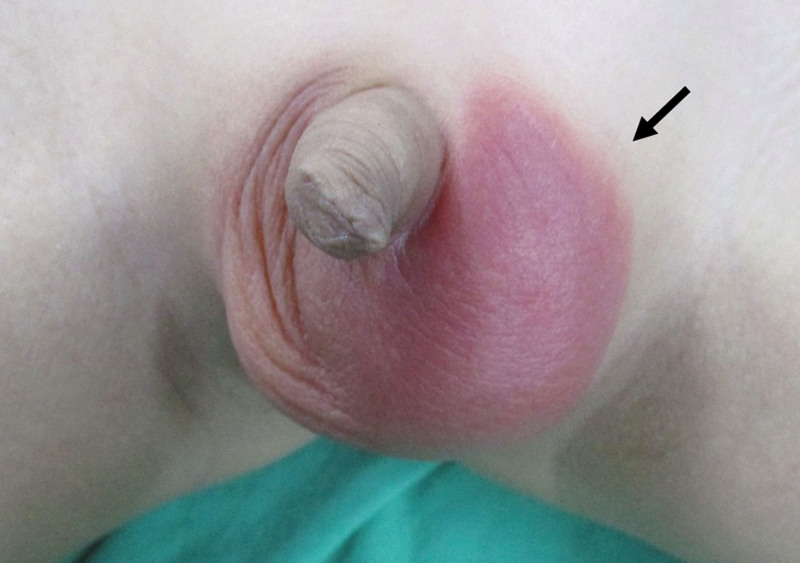
Erythema and swelling of the left scrotum.

**Figure 2.**
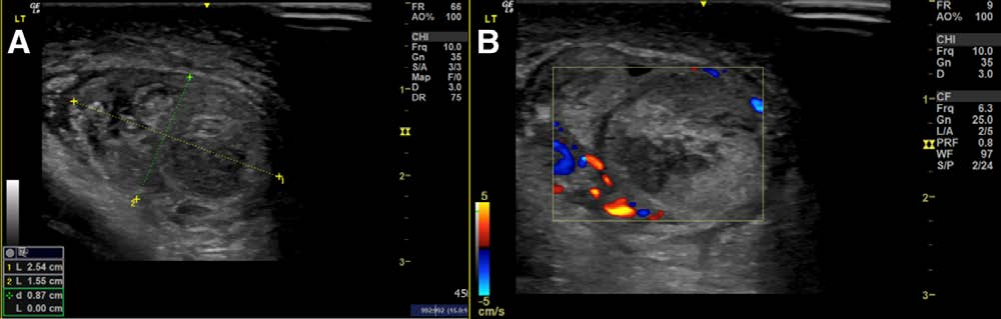
Doppler ultrasound of the left testicle. (A) Heterogenous content of left testicle and (B) no evidence of flow of left testicle.

**Figure 3.**
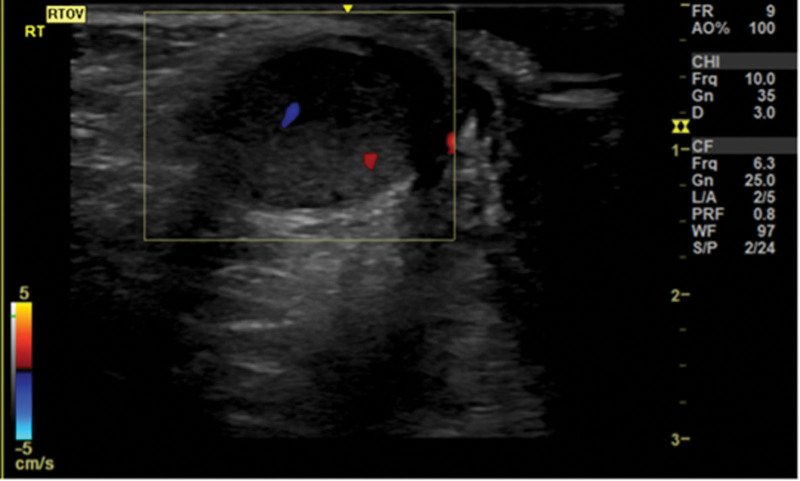
Doppler ultrasound of the right testicle with preserved of flow and homogenous content.

**Figure 4.**
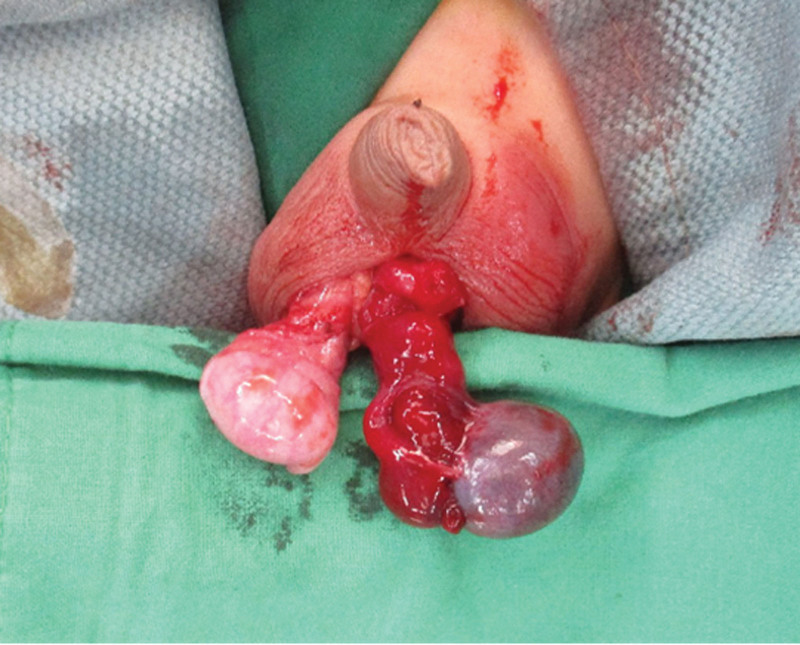
Left testis with necrosis and 720° torsion.

A 7-year-old boy presented to the pediatric emergency Department due to the continuous and progressive left scrotum pain for 2 days. Associated symptoms and signs included left scrotal swelling and erythema (Fig. 1). The patient reported left lower abdominal pain approximately 4 days ago. Initially the patient was suspected to suffer from acute gastroenteritis. The left scrotal pain was not accompanied with nausea or vomiting. No precipitating factors, exacerbating factors and relieving factors were noted. The patient also did not report previous trauma or surgical history. Initially the patient reported having left abdominal pain that migrated to the left scrotum 2 days later, erythema over left scrotum was also present. His family thought it may be caused by scratching, and he was treated symptomatically. The patient was then transferred to the emergency department due to his worsening condition. Physical examination included redness and swelling of the left scrotum with a tender and high-riding testis. It was accompanied by a negative Prehn’s sign and an absent cremasteric reflex on the affected side. Blood laboratory tests revealed no leukocytosis and normal C-reactive protein levels. Color doppler ultrasound of scrotum demonstrated an enlarged, inhomogeneous hypoechoic left testis, and no detectable flow in the left testis (Fig. 2). Blood flow to the right testis was preserved (Fig. 3). These findings pointed towards testicular torsion. Therefore, a surgical intervention within an hour after the patient’s admission was performed. A definitive diagnosis was made due to the findings of a 720° counterclockwise rotation of the spermatic cord and ischemic changes of the left testis and epididymis during the surgery (Fig. 4). However, there was no restoration of blood flow after a 20-minute warm bath and manual detorsion. Therefore, left orchiectomy and right orchiopexy were performed. Furthermore, the patient was empirically prescribed Cephadrine. One day after the operation, fever and abdominal discomfort were complained. Physical examination showed periumbilical mild tenderness, no rebounding pain, and no muscle guarding. Abdominal X ray revealed large stool impaction, no ileus pattern, and no free air. After adequate treatment, symptoms were relieved. The patient was finally discharged in a stable condition on day 5.

## 3. Discussion

Testicular torsion is a relatively common acute scrotal condition in the pediatric emergency Department. The disease happens when there is a twisting of the spermatic cord along its longitudinal axis obstructing venous return^[11,12]^ causing the equalization of venous and arterial pressures resulting in testicular ischemia. Additionally, the reperfusion injury that occurs on the release of the twisted cord adds to the principal pathophysiology of the condition.^[12]^ According to experimental research, testes start to infarct around 2 hours of blood re-striction with irreversible ischemia beginning after 6 hours leading to complete infarction after 24 hours.^[13]^ Contrarily, a systemic study revealed that there is 90% survival of the affected testicular tissue at 0 to 12 hours after the onset of ischemia, half survival at 13 to 24 hours, and up to 18.1% survival after 24 hours.^[14]^ However, Howe et al^[11]^ concluded that 15 hours of symptoms and 860 degrees of torsion gives testes a 50% salvage rate. For maximizing the chances of testicular survival, prompt diagnosis and treatment of testicular torsion is crucial.

Testicular torsion is diagnosed clinically with symptoms of abrupt onset testicular or scrotal pain, scrotal swelling, nausea, and vomiting. Patients may also present with nonspecific symptoms such as fever or urinary problems. Not all cases of testicular torsion present scrotal pain or discomfort. The most common atypical symptoms are lower abdominal pain, inguinal pain, and nausea/vomiting. The lower abdominal pain or inguinal pain may move to the scrotum few hours after the onset of the initial abdominal presentation,^[15,16]^ and the source of pain radiates from scrotal area.^[16]^ Testicular torsion should always be involved in lower abdominal pain’s differential diagnosis in males. Prepubertal children are more likely to present with atypical symptoms than post pubertal children, resulting in delayed diagnosis and intervention.^[17]^ In children presenting with abdominal groin pain and empty ipsilateral scrotum, torsion of undescended testis should be considered.^[18]^ Torsion of cryptorchidism is accounted for about 5% in all testicular torsion cases,^[19]^ and the incidence of torsion of cryptorchidism appears to be higher than torsion of normally descended testis.^[20,21]^ Physical examination is crucial in diagnosing testicular torsion. Unilateral testicular tenderness, high riding testis, trans-verse testicular orientation, anterior palpation of the epididymis, absent cremasteric reflex, and negative Prehn’s sign are some of the key observations.^[22,23]^ Physicians should also examine abdomen or inguinal canal with a tender mass and missing testis in the ipsilateral inguinal region. Testicular Workup for Ischemia and Suspected Torsion (TWIST) score is a useful tool for testicular torsion diagnosis since it encompasses testicular swell-ing, hard testis, nausea/vomiting, absent cremasteric reflex, and high riding testis, which encompasses common signs of testicular torsion.^[24]^ Meta-analyses revealed the TWIST score achieves a high sensitivity (0.984) and high specificity (0.975).^[25]^ However, TWIST does not capture erythema or abdominal pain, highlighting that this case is atypical at first. Point of care ultrasound can be crucial in diagnosis due to its high sensitivity (100%) and specificity (99.1%)^[26]^ and shows relative decrease or absence of blood flow to the affected testis, which significantly increases the predictivity.^[4,27]^

Surgical treatment includes orchiectomy or testicular salvage with detorsion and orchiopexy.^[28]^ Manual detorsion is considered a “time buying procedure” and a pre-liminary to surgical intervention.^[23]^ Manual detorsion should be performed along with surgical exploration and orchidopexy, which remains the gold standard in the diagnosis. Orchiectomy is performed on non-viable testis to prevent the formation of anti-sperm antibodies which may negatively affect the non-affected testis. About 40% of boy undergoing surgery for testicular torsion performed orchiectomy,^[3]^ and rate of orchiectomy is higher in torsion of cryptorchidism than in torsion of descended testis.^[19,29]^ Another study revealed poor rates of surgical salvage in torsion of cryptorchidism.^[30]^ It was because the atypical presentations made parents underestimate the children’s condition. The probability of orchiectomy is highest for children in prepubertal age^[3,17,31]^ and the risk for orchiectomy decreased by 14% each subsequent year.^[17]^ Additionally, orchiopexy is per-formed on viable testis to prevent retorsion,^[32]^ and prophylactic orchiopexy of the unaffected testis should also be performed to prevent future torsion.^[33]^ However, half of the patients undergoing only salvage therapy still develop testicular atrophy within 2 to 14 months after the procedure.^[34]^ Predictive factors include pain lasting over 1 day and heterogeneous echogenicity on ultrasound. Post-op follow up is crucial.

In conclusion, although testicular torsion is less common in the preschool and school attending age group, it is more likely to present with atypical symptoms at this age. Physicians need to perform detailed history taking and physical examination. Point of care ultrasound should be used to promptly diagnose the condition necessary for emergent urologist consultation and intervention to decrease the necessity of orchiectomy and subsequently to prevent testicular loss, testicular atrophy, and eventual impairment of fertility.

## Author contributions

**Data curation:** Bei-Cyuan Guo.

**Methodology:** Bei-Cyuan Guo.

**Resources:** Bei-Cyuan Guo.

**Supervision:** Han-Ping Wu.

**Writing – original draft:** Bei-Cyuan Guo.

**Writing – review & editing:** Han-Ping Wu.
